# Effect of Different Processing Methods on the Millet Polyphenols and Their Anti-diabetic Potential

**DOI:** 10.3389/fnut.2022.780499

**Published:** 2022-02-11

**Authors:** Han Wang, Yongxia Fu, Qingyu Zhao, Dianzhi Hou, Xuehao Yang, Shuqun Bai, Xianmin Diao, Yong Xue, Qun Shen

**Affiliations:** ^1^College of Food Science and Nutritional Engineering, China Agricultural University, Beijing, China; ^2^National Center of Technology Innovation (Deep Processing of Highland Barley) in Food Industry, Beijing, China; ^3^National Engineering Research Center for Fruit and Vegetable Processing, Beijing, China; ^4^Key Laboratory of Plant Protein and Grain Processing, Beijing, China; ^5^Shanxi Institute for Functional Food, Shanxi Agricultural University, Taiyuan, China; ^6^School of Food and Health, Beijing Technology and Business University, Beijing, China; ^7^Cofco Nutrition and Health Research Institute Co., LTD., Beijing, China; ^8^Institute of Crop Science, Chinese Academy of Agricultural Sciences, Beijing, China

**Keywords:** millet, polyphenols, diabetes, processing methods, hypoglycemic

## Abstract

Interest in polyphenols has grown due to their beneficial effect on diabetes attenuation. Millets are ancient crops that are rich in polyphenols and used for both food and feed. They are grown worldwide and are adapted to production under dry, hot conditions. The polyphenols found in millets have anti-diabetic properties. However, millet is usually consumed after being processed by heating, germination, fermentation, and other processing methods, which may alter polyphenol content and thus affect their anti-diabetic potential. This mini-review profiles the effects of different processing methods on millet polyphenols and how changes in millet polyphenols affect the hypoglycemic effect of millet. Future studies are needed to compare the anti-diabetes potential of millet polyphenols before and after processing and to explore ways to minimize polyphenol losses and thus maintain their hypoglycemic effect in final products.

## Introduction

Type 2 diabetes (T2D) is one of the most common metabolic diseases. Various drugs are used to manage diabetes, including biguanides, α-glucosidase inhibitors, and thiazolidinediones. However, long-term use of anti-diabetic drugs can induce side effects such as organ dysfunction and gastrointestinal problems (1, 2). Therefore, new substances are needed to prevent and treat diabetes. Many kinds of cereals have shown beneficial effects on diabetes likely due to the presence of polyphenols. These compounds are a highly heterogeneous group (3). Part of the hypoglycemic effect of polyphenols is due to their inhibitory activity toward α-amylase and α-glucosidase during enzymatic hydrolysis of complex carbohydrates, thus delaying the absorption of glucose and reducing the spike in postprandial blood glucose characteristic of T2D (4–6). In addition, polyphenols could alleviate diabetes by activating the adenosine monophosphate (AMP)-activated protein kinase pathway (7).

Millet, including pearl millet, proso millet, finger millet, foxtail millet, barnyard millet, little millet, and kodo millet, is rich in polyphenols. Millet consumption can be beneficial because these polyphenols have hypoglycemic effects. Although millet is usually eaten after processing by heating, germination, fermentation, and other methods, the effects of processing method on millet polyphenol content has not been systematically reported. Thus, we reviewed the latest research progress on the effect of different processing methods on millet polyphenols and their anti-diabetes potential.

## Polyphenols in Millet

**Figure 1:**
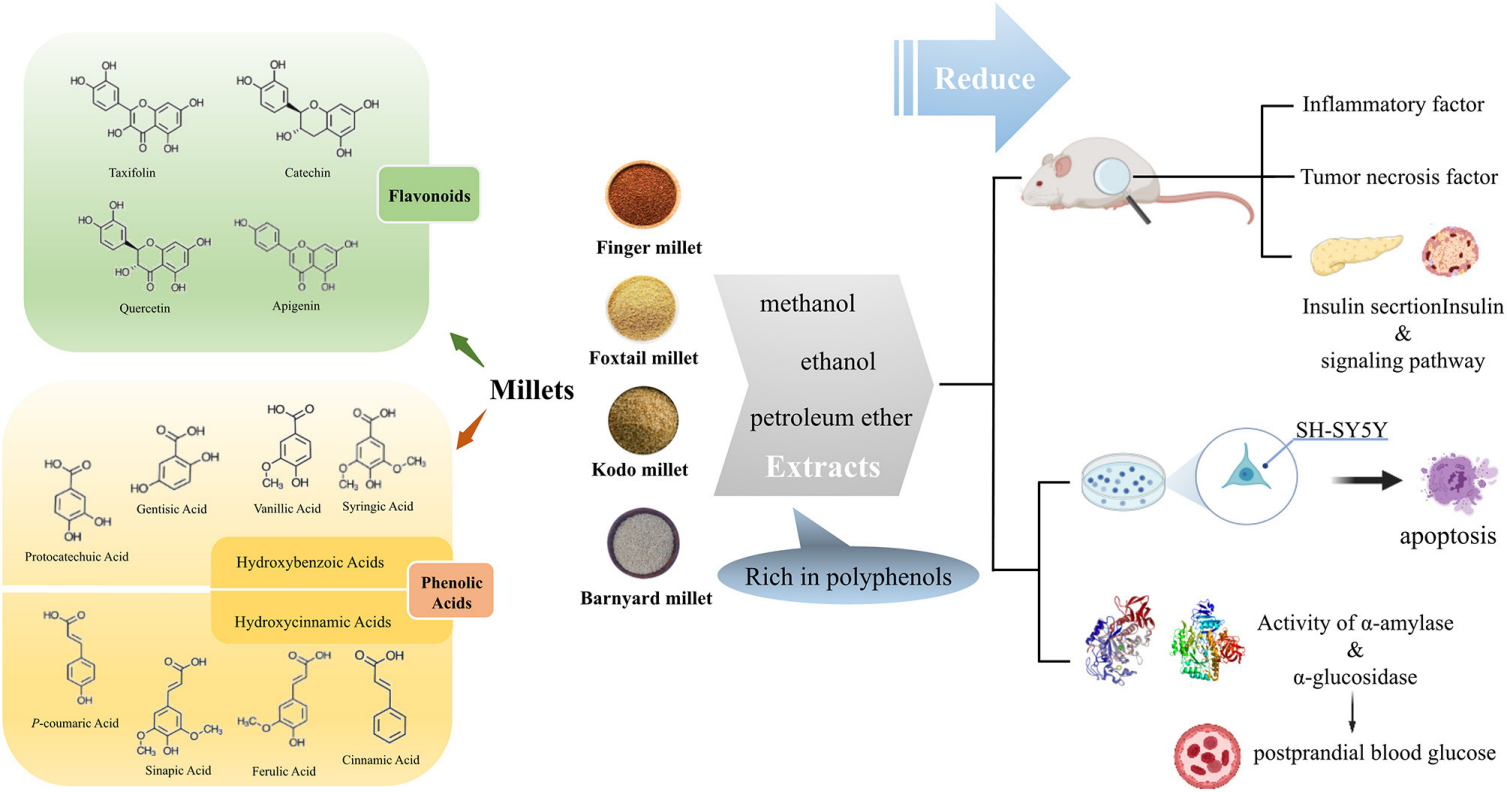
Polyphenols in millets and their effect on diabetes related factors. The polyphenols in millet mainly include phenolic acids and flavonoids. Through the summary of *in vivo* and *in vitro* experiments on the effects of millet polyphenols on diabetes, we found that the polyphenol extracts of millet affected antioxidant and anti-inflammatory factors, the insulin signal pathway, and enzyme activities related to postprandial blood glucose. This figure was partly created with BioRender.com, and the agreement number is IV22Z7AFS9.

Most polyphenols are found within the seed coat of millet (8) and are mainly composed of phenolic acids and flavonoids. The phenolic acids in millet primarily exist in a bound state, including hydroxybenzoic acids and hydroxycinnamic acids. The former contains protocatechuic, gentisic, vanillic, and syringic acids, and the latter mainly includes *p*-coumaric, sinapic, ferulic, and cinnamic acids. In addition, the flavonoids in millet are in the free state and mainly include quercetin, catechin, gallocatechin, taxifolin, and apigenin derivatives (Figure 1) (9). The total contents of polyphenols (TPC), flavonoids (TFC), and tannins varies depending on the millet species (Table 1).

**Table 1:** The total polyphenol content (TPC), the total flavonoids content (TFC) and Tannins in different varieties of millets.

	**TPC**	**TFC**	**Tannins**	**References**
Little millet	429.9 ± 0.40 mg GAE/100 g	334.9 ± 0.89 mg CEQ/100 g	283.4 ± 0.41 mg CEQ/100 g	(10)
	1.51 ± 0.21 mg GAE/g			(11)
Finger Millet	136.4 ± 7.07 mg FAE/100 g	115.8 ± 9.1 mg CEQ/100 g	17.65 ± 3.95 mg CEQ/100 g	(12)
	3.72 ± 0.14 mg GAE/g			(11)
	2.3 ± 0.3 mg GAE/g			(13)
Foxtail millet	33.17 ± 0.15 mg GAE/100 g	28.10 ± 0.19 mg RUE/100 g		(14)
	0.98 ± 0.06 mg GAE/g			(11)
Barnyard Millet	129.5 ± 4.95 mg FAE/100 g	101.3 ± 10.4 mg CEQ/100 g	59.54 ± 4.63 mg CEQ/100 g	(12)
	1.41 ± 0.11 mg GAE/g			(11)
Kodo Millet	4.44 ± 0.15 mg GAE/g			(11)
Proso Millet	1.14 ± 0.11 mg GAE/g			(11)
Pearl Millet	304 ± 0.01 mg GAE/100 g			(15)
	6.58 ± 0.22 mg GAE/100 g			(16)
	51.4 mg GAE/100 g			(17)
	2.15 ± 0.22 mg GAE/g			(11)

*GAE, gallic acid equivalents; CEQ, catechin equivalents; FAE, Ferulic Acid Equivalent; RUE, rutin equivalent. The sample refers to the dry basis*.

Millets are traditionally subjected to different processing methods within the home, including milling, soaking, cooking, roasting, germination, and fermentation (18). These processing methods can change the polyphenol content of the final product. Therefore, we reviewed literature reports on millet processing techniques and their effects on polyphenol content.

## The Effect of Different Processing Methods on Millet Polyphenols

### Fermentation

Fermentation is reported to increase the content of biologically active ingredients and to change the ratio of nutritional to anti-nutritive constituents in millets (19). For instance, fermentation by *Rhizopus azygoporus* significantly increased the TPC in pearl millet from 6.6 to 21.8 mg gallic acid equivalent (GAE)/g on a dry weight basis. Also, the contents of ascorbic acid, p-coumaric acid, gallic acid, and catechol were higher in fermented vs. unfermented pearl millet (16). Yadav et al. treated finger millet using solid-state grain fermentation (SSF) and food-grade biological *Rhizopus oryzae*. On the 6th day, the TPC reached the highest level (18.64 mg GAE/g dry substrate), which was due to the release of phenolics through the activity of carbohydrate cleaving enzymes and β-glucosidase (20). The same trend was also observed in pearl millet fermented by SSF using *Aspergillus sojae* (MTCC-8779). During SSF the production of specific bioactive compounds, including ascorbic, gallic, and p-coumaric acids, significantly increased, which was consistent with results from pearl millet fermented with *R. azygoporus* (16, 21). Similarly, the TPC of dietary fiber in foxtail millet bran significantly increased from 3.05 ± 0.01 mg to 7.02 ± 0.01 mg GAE/g dry matter after fermentation by the *Bacillus natto* (22). To explore the relationship between fermentation time and TPC in millet, Balli et al. used lactobacilli and yeast for fermentation and they found that the TPC reached the highest value after 72 h. They also found that the vitexin and vitexin 2″-O-rhamnoside contents significantly increased after fermentation. These compounds could act as negative regulators of insulin receptors and in turn affect T2D by partially inhibiting the overexpression of protein tyrosine phosphatase-1 B (23). The increase of TPC during fermentation could be caused by the action of microorganisms using soluble and fermentable fiber for their growth and releasing “mechanically trapped” phenolic compounds (PC) from the polymeric fiber structure. However, Gabaza et al. found that fermentation and cooking caused an increase of soluble PC in finger millet, among which catechins increased more than 4-fold. In contrast, the soluble PC, chiefly ferulic, sinapic, and salicylic acids, showed a downward trend (24). The increase of the soluble PC could be due to the reorganization of TPC under low pH during fermentation, resulting in polymerized TPC being transformed into oligomeric polyphenols thus increasing the soluble PC (25–27). The PC of rabadi, a fermented pearl millet food, decreased with increasing fermentation time (28). TPC in finger millet decreased by 26–29% and tannins by 44–52% after fermentation for 48 h in finger millet (29). Moreover, Rani et al. reported that the TPC of foxtail millet decreased as fermentation time increased, and significant changes in catechin were observed during fermentation (30).

### Germination

Germination is part of the malting process, which includes soaking, sprouting, and drying, and can increase the TPC and improve the nutritional value of grain by reducing anti-nutrients like phytates, tannins, and oxalic acid. The optimum germination conditions for the highest TPC (83.01 mg GAE/100 g dry weight basis) and TFC (87.53 mg rutin equivalent (RUE)/g dry weight basis) in kodo millet flour were soaking for 10 h and fermentation at 38.75°C for 35.82 h (31). This procedure also improved the nutritional value of kodo millet flour by decreasing the contents of phytates and tannin by 25 and 85%, respectively. In an optimized germinated kodo millet sample, the major active components as evaluated with gas chromatography-mass spectrometry (GC–MS) were arachidonic amide and pterin-6-carboxylic acid (31). In barnyard millet, Sharma et al. found that the predicted values of TPC and TFC reached their highest values of 54.23 mg GAE/100 g dry weight basis and 42.95 mg RUE/g dry weight basis after soaking for 11.8 h followed by germination for 36.5 h at 33°C. Compared to raw barnyard millet flour, the optimized flour contained higher amounts of hexadecanoic acid (5.59%), octadecadienoic acid (Z, Z), 9,12 octadecadienoic acid, ethyl ester, and other compounds. Among PC, the contents of isomers of phthalic acid and exadecanoic acid were increased significantly (32). Since PC are bound to non-starch polysaccharides in grain cell walls through associations such as ester and ether bonds, the increase in PC during germination in barnyard millet could have resulted from the action of cell wall-degrading enzymes which modified the cell wall structure of the grain and released the PC (27, 32). Germinated millets exhibited the highest TPC compared to millets treated by steaming and microwaving, which was due to an increase in total individual flavonoid content including apigenin and myricetin (33). Another study with finger millet demonstrated that when it was malted for 24 and 48 h, the TPC decreased whereas the content of protocatechuic acid, catechin, and epicatechin increased at 72 and 96 h (34).

### Heat Treatment

Heat treatment refers to roasting, puffing, and parboiling. These treatments improve the eating quality of millets but their effects on nutritional attributes, such as TPC, varies depending on the heating method used.

Roasting time and temperature affect the TPC in millet. For example, the TPC in whole and dehulled proso millet increased from 295 to 670 and 167 to 587 mg FAE /100 g dry weight basis after roasting at 110°C for 10 min (35). Notably, the contents of syringic, gallic, 4 hydroxy benzoic, ferulic, and sinapic acids both in whole and dehulled proso millet significantly increased after roasting. Only the content of catechin showed the opposite trend. The increases could be caused by the hydrolysis of C-glycosyl flavones during roasting promoting the release of polyphenols. However, TPC in finger millet after roasting showed an opposite trend, a decrease from 314.24 mg GAE /100 g dry weight basis to 223.31 mg GAE/100 g dry weight basis (36). And Obadina et al. also found that TPC in pearl millet decreased after roasting at all test temperatures (37).

Puffing and extrusion are usually performed at high temperatures for a short time. Pilat et al. reported that puffed proso millet had a high polyphenol content and thus good nutritional properties (38). The TPC and TFC in extruded finger millet flour were significantly lower than unextruded checks with maximum retention rates of 54 and 78%, respectively (39).

Parboiling can improve the milling yield and physicochemical properties of grains. Bora et al. applied this process to dehulled millet and found that the free and bound polyphenol content of parboiled millet products significantly increased compared with untreated millets. HPLC analysis revealed that the remarkable increase in the content of bound phenolic acids attributable to an increase in ferulic and p-hydroxy acids. This result was probably caused by the partial migration of phenolics from the pericarp to the inner layers of the grain and the release of the cell wall-bound phenolics during parboiling, which would reduce the phenolic loss during dehulling thus increasing the phenolic content in the parboiled millet (40).

The magnitude of the heat treatment effect on polyphenol content at a given temperature depends on the heating method used. When treated at 110°C by either roasting, steaming, puffing, or extrusion, the increase in TPC of proso millet varied with heating method. The highest TPC was obtained by roasting, followed by extrusion, puffing, and steaming, corresponding to 670, 645, 455, and 315 mg ferulic acid equivalent (FAE) /100 g dry weight basis, respectively. Roasting also led to the highest TFC in proso millet (391 mg RUE/100 g dry weight basis), followed by puffing (304 mg RUE/100 g dry weight basis), steaming (282 mg RUE/100 g dry weight basis) and extrusion (219 mg RUE/100 g dry weight basis). The increase of TPC and TFC after roasting may be partially related to the release of bound PC such as ferulic, caffeic, and coumaric acids during heat treatment (35).

### Other Processing Methods

Many other processing methods, including soaking, grinding, shelling, high-pressure, ultrasound, and microwaving, are also used for grain processing. These methods can affect the eating quality characteristics and physicochemical properties of millets and cause changes in polyphenol content.

Dehulling and soaking are commonly used for cereal processing. Millets are usually dehulled before being further processed (41), while soaking can be used to reduce the content of some anti-nutritional ingredients in millets. High-pressure soaking can increase the TPC and decrease contents of anti-nutrients such as phytates and tannins in germinated foxtail millet (42). Apart from dehulling and soaking, milling is often used to maximize endosperm, bran, and germ separation to reduce particle size for further processing. In chapatis, a bread made from pearl millet, when the millet bran was removed from the flour the content of phytates and phenols was substantially reduced. This result was because the phytates and phenols were mainly in the pearl millet bran (43). In addition, ultrasound, enzyme treatment, and the combination of these two can also affect the content of specific polyphenols in millets. Compared to conventional methods, ultrasonication (UA) and UA after enzyme treatment with xylanase (XUA) both increased the extraction rate, respectively, of the TFC 1.4- and 1.3-fold and of the tannin 1.1- and 1.2-fold. In addition, compared with UA, XUA led to a better extraction of phenolic substances in finger millet, which increased 2.3-fold. There were 3-(3″-malonyl) glucoside cyaniding, 6- C-pentosyl-8-C-pentosyl luteolin, and trimers of catechins present in finger millet treated with UA and XUA samples. Moreover, the derivatives of caffeic acid, including caffeoyl shikimic and dicaffeoyl shikimic acids, were only observed in finger millet processed with XUA, which could be the result of xylanase releasing these compounds (44). However, though polyphenols are originally in a stable state in the grain, exogenous enzyme treatment can reduce polyphenol content in millets. Moreover, microwave treatment caused different effects on polyphenol contents in different millets. Pradeep et al. reported that microwave treatment significantly decreased the contents of TPC in barnyard millet yet increased the TPC in foxtail millet and proso millet, gallon acid in foxtail millet, and ferulic, gallic, erucic, and cinnamic acid in proso millet (33). Microwave treatment reduced most of the phenolic acids and total phenolic acid contents in foxtail millet, except for vanillic and erucic acids. The same trend was also observed for caffeic and *p*-coumaric acids in pearl millet.

### The Relationship Between the Hypoglycemic Effect of Millet Polyphenols and Their Content

**Figure 2:**
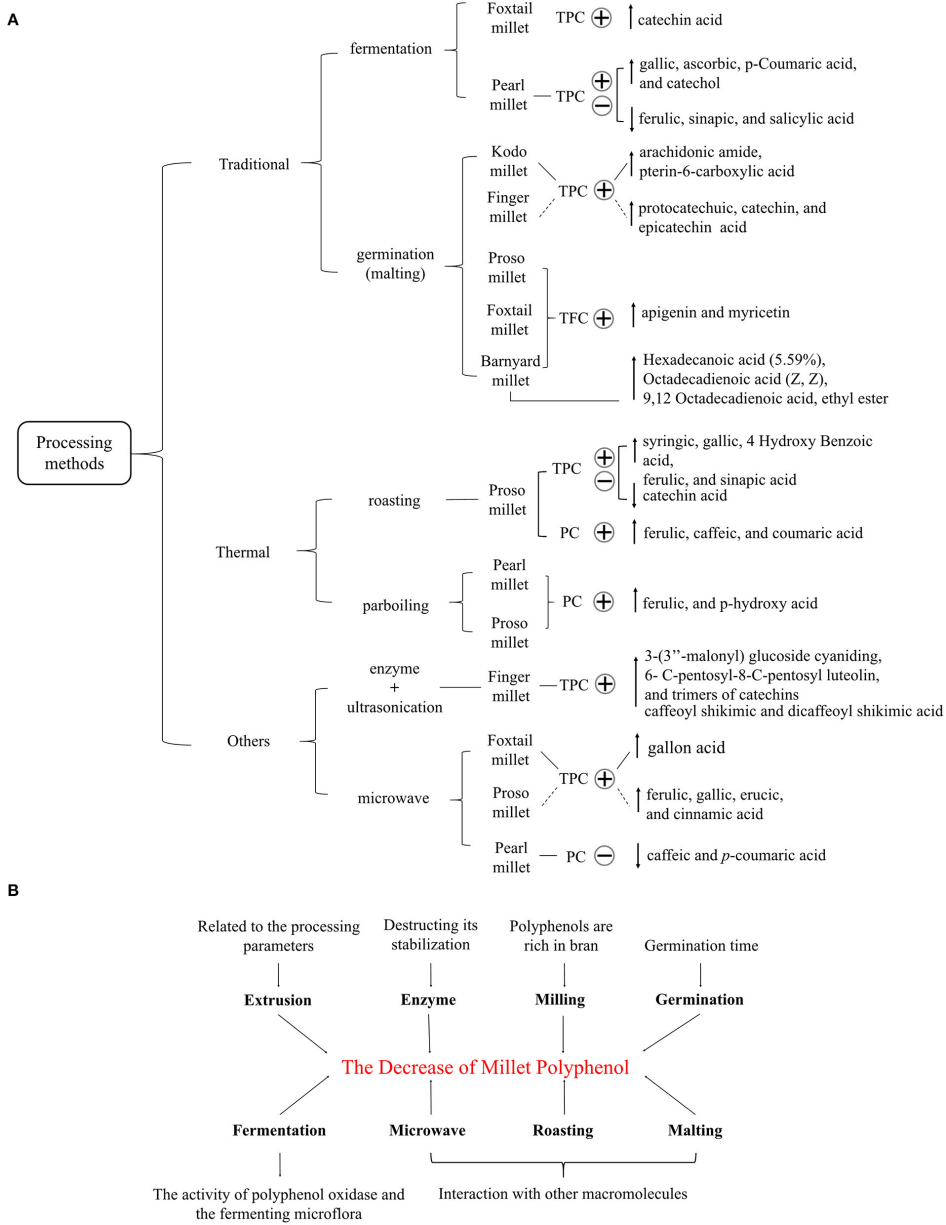
**(A)** The influence of different processing methods on the content of certain polyphenols (PC) and total polyphenols (TPC) in millet. The plus and minus signs represent increase and decrease, respectively. **(B)** The factors related to the decrease of millet polyphenols.

Usually, polyphenols in millets are extracted using organic reagents such as methanol and ethanol. Polyphenols in foxtail millet significantly reduce inflammatory factors in obese rats and alleviate oxidative stress. In addition, the mortality of SH-SY5Y cells (a cell line used to assess insulin resistance) initiated by 300 μM H_2_O_2_ treatment was also decreased by polyphenols in foxtail millet (45) (Figure 1). Polyphenols extracted with methanol from kodo millet and finger millet markedly reduced liver lipid levels and prevented the overexpression of gluconeogenesis genes in the liver of obese Swiss albino mice. These compounds also improved insulin resistance, as evidenced by the increase of the homeostasis model assessment–insulin resistance index. However, only the kodo millet polyphenol extracts significantly increased the quantitative insulin sensitivity check index. Further analysis indicated that the extracts of finger millet chiefly included catechin, vanillic, chlorogenic, and protocatechuic acids whereas taxifolin acid was the main phenolic in kodo millet extracts (46). In addition, Ofosu et al. reported that ethanol extracts containing soluble polyphenols of finger millet significantly increased inhibitory activity (IC50 = 18.07 μg/ml). This effect on α-glucosidase also decreased formation of advanced glycation end-products (AGEs) (12), a key pathophysiological event related to the onset and progression of diabetes. Moreover, oxidation of glycated collagen may be partially responsible for collagen crosslinking and attendant complications in diabetes mellitus. The methanolic extract of finger millet and kodo millet protected collagen from glycation, thus possibly alleviating a complication of diabetes (47). Furthermore, N-p-coumaroyl serotonin, feruloyl serotonin, and luteolin in the polyphenols from barnyard millet effectively inhibited α-glucosidase activity (48). Therefore, polyphenols in millet can positively affect glucose metabolism disorders and contribute to glucose homeostasis (49, 50). The polyphenols in millet change with different processing methods and direction of the effect varies with the type of millet (Figure 2A). Overall, fermentation, germination, heat treatment, and other processing methods can increase the TPC in millets, thus enhancing the anti-diabetic potential.

Previous research demonstrated the potential role of oxidative stress in T2D, which is related to the progression of insulin resistance and β-cell dysfunction both in pre-diabetes and in the clinical phase of the disease (51). Therefore, reducing oxidative stress through increased intake of antioxidants could help alleviate T2D. Polyphenols are nonenzymatic antioxidants with good antioxidant capacity. However, the antioxidant activity of millet polyphenols can change depending on processing method. For example, the contents of ascorbic, gallic, and p-coumaric acids in pearl millet increased remarkably with fermentation, thus enhancing the antioxidant activity of pearl millet extracts (21). Germination, steaming, and roasting all increased the Fe3^+^ reducing power of little millet polyphenols, mainly ferulic acid, thus suggesting an enhancement of antioxidant activity (10). Germinated millets showed the highest phenolic content as well as superior antioxidant and enzyme inhibitory activity compared to millet processed by other methods (31). In addition, Sharma et al. demonstrated that germination increased the *in vitro* antioxidant activity of millet flour extracts, and this effect was attributed to the elevation of phenolics, flavonoids, and γ-aminobutyric acid (GABA) content (14, 31). Azad et al. found that heat treatments including roasting, steaming, puffing, and extrusion increased the total antioxidant capacity of millet via an increase in millet polyphenols (35). The higher flavonoid content in roasted millet modified eicosanoid biosynthesis, thereby resisting inflammation, and protecting low-density lipoprotein from oxidation (35). Processing methods can also induce the production of new polyphenols, thus affecting the antioxidant capacity of millet extracts. For instance, in germinated foxtail millet, the increase of DPPH and hydrogen peroxide scavenging activities were the result of an increase in hexadecanoic acid methyl ester and 9,12-octadecadienoic acid ethyl ester, and the synthesis of new compounds such as pentadecanoic acid and 14-methyl-methyl ester (14). Also, *p*-coumaric acid was produced during parboiling, which may account for the significant increase in the DPPH activity of millet extracts (40). In summary, the enhancement of the antioxidant activity of millet polyphenols after processing can benefit glucose metabolism when these products are consumed.

However, these processing methods sometimes cause a decrease in the content of polyphenols in millets (Figure 2B). For example, a reduction in polyphenols during fermentation may result from the activity of polyphenol oxidase and the fermenting microflora (26). A rearrangement of phenolic structures due to the acidic environment during fermentation and a decrease in the extractability of PC due to self-polymerization can affect the TFC of the millet flours (52). In addition, malting, fermentation, and thermal processing caused a decrease in bound phenolic compounds in millets by through the reorganization of TPC and its interaction with other macromolecules such as proteins (27). The reduction of polyphenol extraction after roasting and microwaving may be related to the formation of conjugates with free acids and to complexing with proteins, tannins, and polyphenols such as anthocyanins (33, 41). Furthermore, enzyme treatment decreased polyphenol content by hydrolyzing other substances in millets, thus destroying polyphenol stability. The reduction of polyphenols by milling was due to the removal of phytates and phenols with the removal of the millet bran (26). Processing parameters during germination and extrusion such as time, moisture, temperature, and screw speed can also affect polyphenol content (34, 38). Consequently, a reduction of polyphenols weakens antioxidant activity and thus reduces the hypoglycemic effect of the final product.

### Future Direction

Many studies to date have only focused only on the changes in the content and antioxidant activity of polyphenols before and after processing. Few comparative studies have been done on how the changes in polyphenols before and after processing affect diabetes. The reduction of polyphenols during processing could affect any beneficial effects on diabetes. In addition, though polyphenols like catechins and ferulic acid are known for their antioxidant, hypoglycemic, and anti-inflammatory effects, other trace polyphenols and their derivatives in millets also need to be studied to understand their potential benefits for human health.

Since phenolic compounds in millets are beneficial, it is necessary to minimize their loss during processing. At present, there is a lack of research on how to mitigate the loss of phenolic substances during processing and this area needs further exploration.

Finally, Folin-Ciocalteu colorimetry is commonly used for quantifying the TPC. This method is obsolete because the structure and physicochemical properties of the macromolecules present in millet change during processing, thus affecting the TPC. For example, the combination of proteins and fibers with phenolics in raw millets may change during processing, thus influencing in the accuracy of TPC evaluation. Although HPLC, GC-MS, and other techniques have been adopted to quantify the contents of certain phenolics, further improvements in the assay for the TPC are needed to obtain more accurate results.

## Conclusion

Millet polyphenols contents changed after processing by various methods and these changes in turn affect the antioxidant and hypoglycemic properties of final products. Considering the great potential of millet polyphenols as anti-diabetic compounds, developing processing methods to maintain and maximize the beneficial attributes of polyphenols should be a focus of future research.

## Author Contributions

All authors listed have made a substantial, direct, and intellectual contribution to the work and approved it for publication.

## Funding

This work was financially supported by China Agriculture Research System of MOF and MARA (CARS-06-13.5) and Cooperation project between China Agricultural University and Datong City [201904710611627].

## Conflict of Interest

XY and SB were employed by Cofco Nutrition and Health Research Institute Co., LTD. The remaining authors declare that the research was conducted in the absence of any commercial or financial relationships that could be construed as a potential conflict of interest.

## Publisher's Note

All claims expressed in this article are solely those of the authors and do not necessarily represent those of their affiliated organizations, or those of the publisher, the editors and the reviewers. Any product that may be evaluated in this article, or claim that may be made by its manufacturer, is not guaranteed or endorsed by the publisher.
