# Corrigendum: Effect of Different Processing Methods on the Millet Polyphenols and Their Anti-diabetic Potential

**DOI:** 10.3389/fnut.2022.879470

**Published:** 2022-03-23

**Authors:** Han Wang, Yongxia Fu, Qingyu Zhao, Dianzhi Hou, Xuehao Yang, Shuqun Bai, Xianmin Diao, Yong Xue, Qun Shen

**Affiliations:** ^1^College of Food Science and Nutritional Engineering, China Agricultural University, Beijing, China; ^2^National Center of Technology Innovation (Deep Processing of Highland Barley) in Food Industry, Beijing, China; ^3^National Engineering Research Center for Fruit and Vegetable Processing, Beijing, China; ^4^Key Laboratory of Plant Protein and Grain Processing, Beijing, China; ^5^Shanxi Institute for Functional Food, Shanxi Agricultural University, Taiyuan, China; ^6^School of Food and Health, Beijing Technology and Business University, Beijing, China; ^7^Cofco Nutrition and Health Research Institute Co., LTD., Beijing, China; ^8^Institute of Crop Science, Chinese Academy of Agricultural Sciences, Beijing, China

**Keywords:** millet, polyphenols, diabetes, processing methods, hypoglycemic

In the published article, there were errors in the affiliations for “Yongxia Fu” and “Dianzhi Hou.” Instead of “Yongxia Fu^1, 2, 3, 4, 5, 6^” and “Dianzhi Hou^1, 2, 3, 4, 5, 6^” it should be “Yongxia Fu^5^” and “Dianzhi Hou^6^.”

In addition, there was an error in affiliation 5. Instead of “Shanxi Institute for Functional Food, Shanxi Agricultural University, Jinzhong, China,” it should be “Shanxi Institute for Functional Food, Shanxi Agricultural University, Taiyuan, China.”

In the original article, there were also grammatical errors in the last sentence of the caption for **Figure 1**. The correct caption appears below:

**Figure 1**. Polyphenols in millets and their effect on diabetes related factors. The polyphenols in millet mainly include phenolic acids and flavonoids. Through the summary of *in vivo* and *in vitro* experiments on the effects of millet polyphenols on diabetes, we found that the polyphenol extracts of millet affected antioxidant and anti-inflammatory factors, the insulin signal pathway, and enzyme activities related to postprandial blood glucose. This figure was partly created with BioRender.com, and the agreement number is IV22Z7AFS9.

The authors apologize for this error and state that this does not change the scientific conclusions of the article in any way. The original article has been updated.

## Publisher's Note

All claims expressed in this article are solely those of the authors and do not necessarily represent those of their affiliated organizations, or those of the publisher, the editors and the reviewers. Any product that may be evaluated in this article, or claim that may be made by its manufacturer, is not guaranteed or endorsed by the publisher.

